# Comparative outcomes of ureteroscopy and percutaneous nephrolithotomy in CKD patients with renal calculi: a propensity-matched cohort study

**DOI:** 10.3389/fmed.2025.1644526

**Published:** 2025-10-03

**Authors:** Jiun-Kai Chiou, Jheng-Yan Wu, Yu-Min Lin

**Affiliations:** ^1^Department of Urology, Taichung Veterans General Hospital, Taichung, Taiwan; ^2^Department of Nutrition, Chi Mei Medical Center, Tainan, Taiwan; ^3^Department of Public Health, College of Medicine, National Cheng Kung University, Tainan, Taiwan; ^4^Division of Cardiology, Department of Internal Medicine, Chi Mei Medical Center, Chiali, Tainan, Taiwan

**Keywords:** CKD, MAKE, PCNL, renal stone, URS

## Abstract

**Background:**

Kidney stones frequently coexist with chronic kidney disease (CKD), sharing common risk factors and leading to adverse outcomes. While percutaneous nephrolithotomy (PCNL) and ureteroscopy (URS) are both effective treatment options, the safety of PCNL has been well-established in CKD patients, whereas the safety profile of URS remains less clear.

**Methods:**

A retrospective cohort study using the TriNetX database was conducted to compare outcomes in CKD patients undergoing URS or PCNL. Patients aged ≥18 years with a diagnosis of renal stones and CKD were included, excluding those on dialysis. Propensity score matching (PSM) was performed to balance baseline characteristics. The primary outcome was the 5-year rate of major adverse kidney events (MAKE), a composite of mortality, dialysis initiation, and worsened renal function. Secondary outcomes included all-cause mortality and dialysis dependence. Subgroup and sensitivity analyses were performed to ensure robustness.

**Results:**

Of 5,470 eligible patients, 837 underwent URS and 4,633 underwent PCNL, with 723 patients matched in each group post-PSM. There was no significant difference in MAKE between URS and PCNL (HR 0.93; 95% CI 0.68–1.28; *p* = 0.6952). All-cause mortality (HR 0.98; 95% CI 0.70–1.36; *p* = 0.9125) and dialysis dependence (HR 0.57; 95% CI 0.23–1.38; *p* = 0.2128) were also similar. The limitation of this study is the lack of data on stone size and location.

**Conclusion:**

In CKD patients with renal stones, URS demonstrated comparable safety and efficacy compared to PCNL, with no significant differences in MAKE, all-cause mortality, or dialysis dependence over 5 years. However, the lack of information regarding stone size, anatomical location, and procedure-specific details (e.g., device use or surgical technique) is a major limitation of our study.

## Introduction

Kidney stones are one of the most common kidney diseases, with a rising prevalence worldwide (21.11%) (1). The management of renal stones primarily involves percutaneous nephrolithotomy (PCNL) and ureteroscopy (URS). PCNL is the first-line procedure for renal calculi larger than 20 mm, as it provides the advantage of a higher stone-free rate and is not constrained by stone burden or composition (2, 3). Unfortunately, an international multicenter study involving 5,803 patients undergoing PCNL reported an overall complication rate of 21.5%, including bleeding, sepsis, renal insufficiency, mortality, and so forth (4, 5). URS, a relatively newer technique for managing urolithiasis, is often preferred due to its higher stone-free rate compared to shockwave lithotripsy and its lower complication rate compared to PCNL (6). The complications associated with URS include bleeding, perforation, and renal injury, among others. The overall complication rate for URS ranges from 9 to 21%, with the majority being minor and not requiring intervention (7, 8).

Kidney stones and CKD share several common risk factors, including inadequate fluid intake, bacterial infections, and urinary tract anomalies, which may contribute to their frequent coexistence (9). Moreover, urolithiasis has been associated with a higher risk of developing CKD compared to individuals without urolithiasis (10). Patients with concomitant CKD and renal stones exhibit worse prognoses and elevated surgical risks, highlighting the need for greater attention to this population (11, 12). Both URS and PCNL are effective methods for renal stone removal; however, they may cause renal parenchymal damage and further deteriorate renal function (13–15). A systematic review has demonstrated that PCNL is safe and effective for patients with CKD (11). Additionally, an observational study involving 60 patients supports the conclusion that PCNL is safe and effective for CKD patients (12). On the other hand, URS is considered a less invasive procedure, potentially offering a viable alternative for those patients with higher surgical risks. Although multiple observational studies suggest that URS could be a potential treatment option for renal stones in CKD patients, their conclusions are limited by small sample sizes and a lack of long-term follow-up data (16, 17).

It remains unclear whether URS is as safe as PCNL in patients with CKD and renal stones. To address this knowledge gap, our study utilizes the TriNetX database to compare outcomes between patients undergoing URS and PCNL, focusing on critical endpoints such as mortality and the need for dialysis in 5 years.

## Methods

### Data source

This retrospective cohort study utilized the TriNetX database, which compiles de-identified patient-level data derived from electronic health records. The database gathers information from healthcare organizations (HCOs), primarily academic medical centers, encompassing their main hospitals, affiliated satellite hospitals, and outpatient clinics. The data include patient demographics, diagnoses [using the International Classification of Diseases, Tenth Revision, Clinical Modification (ICD-10-CM)], procedures [coded via the ICD-10 Procedure Coding System or Current Procedural Terminology (CPT)], medications (categorized by the Veterans Affairs Drug Classification System and RxNorm codes), laboratory tests [identified through Logical Observation Identifiers Names and Codes (LOINC)], and healthcare utilization metrics. For this study, we accessed data from TriNetX’s Global Collaborative Network, which spans over 124 million patients across 131 HCOs in 15 countries (18).

The results were validated using industry-standard methodologies and presented in summarized form. Further details about the database are available online (19) and in prior publications. The results were validated using independent, industry-standard methods, and provided to investigators in a summarized format. Further details about the database are available online (19) and have been previously described in the literature (18).

Since the study utilized only aggregated statistical summaries of de-identified data, the need for informed consent was waived. The study adhered to the ethical principles of the Declaration of Helsinki (20) and complied with the Strengthening the Reporting of Observational Studies in Epidemiology (STROBE) reporting guidelines (21). Notably, information regarding stone size, composition, and anatomical location was not available in the TriNetX database. This limitation should be considered when interpreting the study findings.

### Ethics approval and consent to participate

The Western Institutional Review Board granted a waiver for informed consent, citing TriNetX’s capability to generate only aggregated and statistical summaries from de-identified data obtained from multiple healthcare providers. This approach ensures the protection of patient privacy and confidentiality. As the study relied solely on aggregated statistical summaries of de-identified data, informed consent was deemed unnecessary and subsequently waived. As per institutional and regulatory guidelines, studies using such data are exempt from Institutional Review Board approval.

### Cohort

The study included patients aged 18 years or older with a diagnosis of renal stones and CKD. Patients who have already undergoing dialysis were excluded. Participants who underwent procedures for renal stones were categorized into two groups: those treated with URS and those treated with PCNL. For this study, the database was last accessed on November 16, 2024. The index event was the date of the URS or PCNL treatment. Both groups were followed for up to 5 years. Supplementary Table 1 provides detailed information on the codes used to identify demographics, diagnoses, and laboratory parameters.

### Covariables

A 1:1 propensity score matching (PSM) was performed using 25 variables, including demographics, diagnoses, and laboratory data. Covariate selection was informed by clinical relevance, prioritizing major comorbidities and risk factors known to influence renal failure and mortality (22). The selected variables were used to address baseline imbalances between the URS and PCNL groups. These variables encompassed (1) age, sex, race, and ethnicity; (2) comorbidities included essential hypertension, disorder of lipoprotein metabolism, diabetes mellitus, overweight and obesity, ischemic heart disease, heart failure, cerebrovascular disease, calculus in bladder, hyperparathyroidism and disorder of parathyroid gland, hyperparathyroidism, systemic lupus erythematosus, and uric acid nephrolithiasis; and (3) laboratory data such as estimated glomerular filtration rate, calcium, phosphate, magnesium, parathyrin intact, and urate level. Additional information on the categorization and codes used to define the covariates can be found in Supplementary Table 1.

### Primary and secondary outcomes

The primary outcome was the cumulative incidence of major adverse kidney event (MAKE), which was defined as mortality, initiation of dialysis, or worsened renal function. Secondary outcomes included dialysis dependence and all-cause mortality. Patients were followed from the day after the index date for up to 5 years.

### Subgroup and sensitivity analysis

We conducted prespecified subgroup analyses based on age (≥65 or <65 years), gender (male or female), patients with advanced chronic kidney disease (estimated glomerular filtration rate <45 mL/min/1.73m^2^) and the presence or absence of diabetes mellitus, overweight and obesity, heart failure, and coronary artery disease to determine whether the outcomes were consistent across different populations.

To evaluate the robustness of our findings, we conducted a sensitivity analysis by changing the follow-up period to one and 2 years and removing PSM analysis.

### Statistical analysis

The baseline characteristics of the two groups were summarized as means with standard deviations (SDs) for continuous variables and as counts with percentages for categorical variables. Comparisons of categorical variables were performed using the *χ*^2^ test, while continuous variables were analyzed with an independent two-sample *t*-test. One-to-one PSM was carried out using the greedy nearest neighbor algorithm with a caliper width of 0.1 pooled SDs to ensure balance in baseline characteristics between the groups. Matching was considered adequate if the standardized difference between the groups was less than 0.1 (Table 1) (23).

**Table 1:** Baseline characteristics of the URS and PCNL groups before and after propensity score matching.

Characteristics	Before matching, no. (%)			After matching, no. (%)		
	URS (*n* = 837)	PCNL (*n* = 4,633)	Standardized difference	URS (*n* = 723)	PCNL (*n* = 723)	Standardized difference
Age, mean(SD), years	60.6 ± 15.9	61.1 ± 14.8	0.0329	60.5 ± 15.9	60.0 ± 15.7	0.0332
Sex						
Female	368 (46.8%)	2,186 (48.7%)	0.0368	339 (46.8%)	339 (46.8%)	<0.0001
Male	396 (50.4%)	2,215 (49.3%)	0.0216	364 (50.3%)	369 (51.0%)	0.0138
Race						
White	566 (72.1%)	3,487 (77.7%)	0.1297	565 (78.1%)	544 (75.2%)	0.0687
Black or African American	46 (5.8%)	359 (8.0%)	0.0844	46 (6.3%)	47 (6.5%)	0.0056
Unknown race	146 (18.5%)	345 (7.6%)	0.3272	85 (11.7%)	107 (14.7%)	0.0898
Asian	12 (1.5%)	137 (3.0%)	0.1020	12 (1.6%)	10 (1.3%)	0.0226
Comorbidities						
Essential hypertension	409 (52.1%)	2,456 (54.7%)	0.0528	385 (53.2%)	389 (53.8%)	0.0111
Disorder of lipoprotein metabolism	209 (26.6%)	1,804 (40.2%)	0.2909	209 (28.9%)	196 (27.1%)	0.0401
Diabetes mellitus	238 (30.3%)	1,479 (32.9%)	0.0569	219 (30.2%)	207 (28.6%)	0.0364
Overweight and obesity	186 (23.6%)	1,173 (26.1%)	0.0566	174 (24.0%)	162 (22.4%)	0.0393
Ischemic heart disease	130 (16.5%)	839 (18.6%)	0.0561	123 (17.0%)	113 (15.6%)	0.0374
Heart failure	68 (8.6%)	461 (10.2%)	0.0551	64 (8.8%)	59 (8.1%)	0.0248
Cerebrovascular disease	52 (6.6%)	383 (8.5%)	0.0723	50 (6.9%)	53 (7.3%)	0.0161
Calculus in bladder	50 (6.3%)	341 (7.6%)	0.0483	48 (6.6%)	42 (5.8%)	0.0344
Hyperparathyroidism and disorder of parathyroid gland	20 (2.5%)	150 (3.3%)	0.0470	20 (2.7%)	14 (1.9%)	0.0548
Hyperparathyroidism, unspecified	13 (1.6%)	116 (2.5%)	0.0645	13 (1.7%)	10 (1.3%)	0.0332
Systemic lupus erythematosus	10 (1.2%)	38 (0.8%)	0.0417	10 (1.3%)	10 (1.3%)	<0.0001
Uric acid nephrolithiasis	10 (1.2%)	59 (1.3%)	0.0036	10 (1.3%)	10 (1.3%)	<0.0001

Characteristics	Before matching, mean ± SD			After matching, mean ± SD		
	URS (*n* = 837)	PCNL (*n* = 4,633)	Standardized difference	URS (*n* = 723)	PCNL (*n* = 723)	Standardized difference
Laboratory data						
Estimated glomerular filtration rate, mL/min/1.73 m^2^	57.9 ± 30.7	58.2 ± 27.4	0.0107	57.9 ± 30.7	59.2 ± 32.9	0.0411
Calcium, mg/dL	9.0 ± 0.7	9.2 ± 0.6	0.3123	9.0 ± 0.7	9.3 ± 0.7	0.3519
Phosphate, mg/dL	3.3 ± 0.7	3.3 ± 0.8	0.0150	3.3 ± 0.7	3.2 ± 0.7	0.1539
Magnesium, mg/dL	1.8 ± 0.2	1.8 ± 0.2	0.0400	1.8 ± 0.2	1.8 ± 0.2	0.0414
Parathyrin intact, pg/mL	69 ± 48.1	76.8 ± 81.8	0.1162	68.6 ± 48.2	70.5 ± 53.6	0.0370
Urate, mg/dL	6.35 ± 1.8	6.45 ± 2.2	0.0503	6.35 ± 1.8	6.56 ± 2.9	0.0848

URS, ureteroscopy; PCNL, percutaneous nephrolithotomy; SD, standardized differences.

Survival probabilities after PSM were estimated using the Kaplan–Meier method and log-rank tests. Hazard ratios (HRs) with 95% confidence intervals (CIs) and *p*-values were determined using Cox proportional hazards regression models for all outcomes. The *E*-value method was applied to assess the potential influence of unmeasured confounding, estimating the minimum strength of association an unmeasured confounder would require to account for the observed differences between the two groups. An *E*-value of x indicates that the observed association could only be attributed to an unmeasured confounder if it were associated with both the treatment and the outcome by a risk ratio of at least x, beyond the effects of the measured confounders (Table 2) (24).

**Table 2:** Comparison of URS vs. PCNL for primary and secondary outcomes.

Outcome	Number of patients with outcomes		HR (95%CI)	*p*-value	*E*-value
	URS (*n* = 723)	PCNL (*n* = 723)			
Primary outcome					
Major adverse kidney events	79	77	0.93 (0.68–1.28)	0.6952	1.36
Secondary outcome					
Dialysis dependence	10	13	0.57 (0.23–1.38)	0.2128	2.90
All-cause mortality	75	70	0.98 (0.70–1.36)	0.9125	1.16

URS, ureteroscopy; PCNL, percutaneous nephrolithotomy; HR, hazard ratio.

All statistical tests were two-sided, with a significance threshold set at *p* < 0.05. Statistical analyses were conducted using the analytic tools available on the TriNetX platform.

## Results

**Figure 1:**
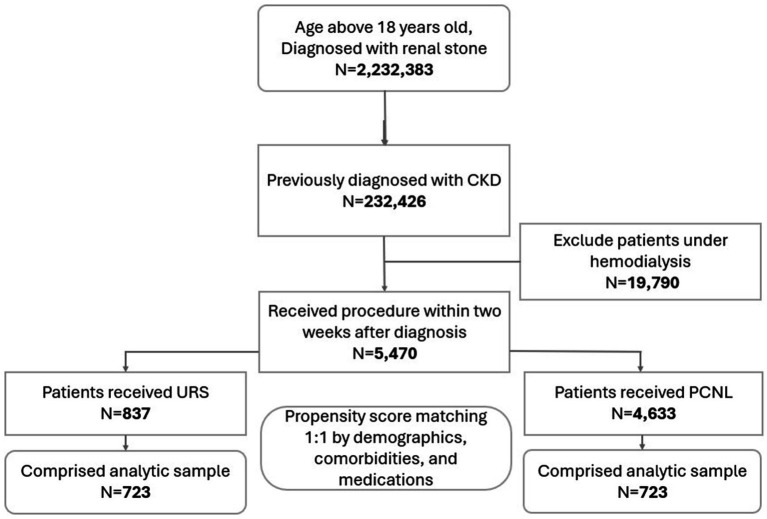
Flow diagram of cohort construction. CKD, chronic kidney disease; URS, ureteroscopy; PCNL, percutaneous nephrolithotomy.

A total of 2,232,383 patients aged over 18 were diagnosed with renal stones. Among them, 232,426 had a prior diagnosis of chronic kidney disease. After excluding patients undergoing hemodialysis, 5,470 individuals underwent URS or PCNL within 2 weeks of their renal stone diagnosis. Of these, 837 patients received URS, while 4,633 received PCNL. Based on demographics, comorbidities, and laboratory data, PSM resulted in 723 patients in each group (Figure 1).

Before PSM, there were no significant differences in age (60.6 ± 15.9 vs. 61.1 ± 14.8, *p* = 0.3827) or the proportion of males (50.4% vs. 49.3%, *p* = 0.5762) between the URS and PCNL groups. However, the URS group had a lower percentage of White (72.1% vs. 77.7%, *p* = 0.0006), African American (5.8% vs. 8.0%, *p* = 0.0377), and Asian patients (1.5% vs. 3.0%, *p* = 0.0174) compared to the PCNL group. Patients who underwent URS also had a lower prevalence of dyslipidemia (26.6% vs. 40.2%, *p* < 0.0001) compared to those in the PCNL group. Other comorbidities were similar between the two groups. Regarding laboratory data, the URS group had a lower serum calcium level (9.06 ± 0.7 vs. 9.28 ± 0.6, *p* < 0.0001) compared to the PCNL group, while other laboratory parameters were comparable between the groups. After matching, the baseline characteristics were balanced and showed no significant differences between the groups (*p* > 0.05) (Table 1).

The propensity score density curves before and after matching are presented in Supplementary Figure 1.

### Primary outcome

**Figure 2:**
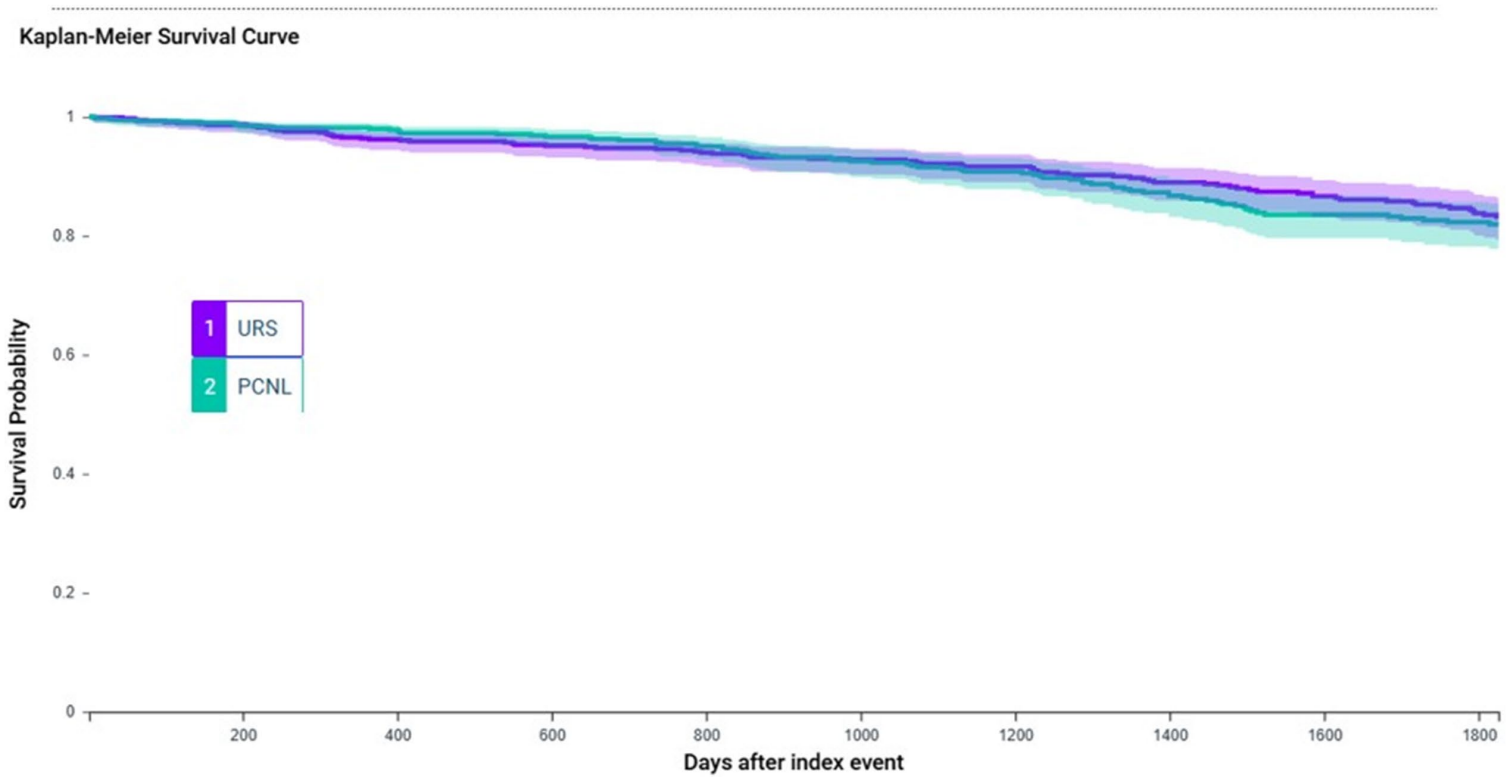
This Kaplan–Meier survival curve compares patients who received URS versus PCNL. There is no significant difference in 5 year MAKE rate between two groups. URS, ureteroscopy; PCNL, percutaneous nephrolithotomy; MAKE, major adverse kidney events.

Over the 5-year follow-up period, 79 (10.9%) patients experienced MAKE in the URS group, and 77 (10.7%) individuals in PCNL group. There is no significant difference between URS and PCNL group [HR 0.93; 95% CI 0.68–1.28; *p* = 0.6952 (Table 2 and Figure 2)].

### Secondary outcome

The risk of dialysis dependence (HR 0.57; 95% CI 0.23–1.38; *p* = 0.2128) and all-cause mortality (HR 0.98; 95% CI 0.70–1.36; *p* = 0.9125) showed no statistical difference between patients who received URS and PCNL (Table 2). Based on the *E*-value analysis, it is improbable that unmeasured confounders had a substantial impact on our results. The *E*-values for the point estimates were 1.36 for MAKE, 2.90 for dialysis dependence, and 1.16 for all-cause mortality, respectively (Table 2).

### Subgroup and sensitivity analysis

The results of this study were consistent across subgroups, including age (≥65 or <65 years), sex (male or female), patients with advanced CKD (estimated glomerular filtration rate <45 mL/min/1.73 m2) and the presence or absence of diabetes mellitus, obesity, heart failure, and coronary artery disease (Supplementary Table 2).

In sensitivity analyses, the outcomes remained non-significant at both the 1-year and 3-year follow-ups and were consistent in analyses without PSM (Supplementary Table 2).

## Discussion

Our research revealed no significant difference in the 5-year MAKE cumulative incidence rate between URS and PCNL in patients with CKD. Similarly, the secondary outcomes showed no significant differences in 5-year all-cause mortality or dialysis dependence between the two groups. These findings were consistent across subgroups stratified by age, sex, advanced CKD, and the presence or absence of diabetes mellitus, obesity, heart failure, or coronary artery disease. Sensitivity analyses confirmed these results at 1-and 3-year follow-ups, as well as in analyses without PSM.

Clinical diagnosis of CKD has been found to be more prevalent in kidney stone formers compared to control subjects, highlighting the potential impact of kidney stones on renal function (25). This association may be due to repeated episodes of obstructive uropathy, infections, or inflammation caused by stones, which can lead to progressive kidney damage and scarring (9). The coexistence of CKD and renal stones is associated with a significantly worse prognosis compared to patients without CKD (11). CKD patients are also more vulnerable to surgical complications and slower recovery (26) Moreover, the presence of both conditions may accelerate the progression of renal function decline, increasing the risk of end-stage kidney disease, and mortality (11, 12, 27). These findings underscore the importance of early detection and management of kidney stones to mitigate the risk of CKD progression.

Previous studies have demonstrated the safety and effectiveness of PCNL in CKD patients, highlighting its ability to achieve high stone-free rates while maintaining acceptable complication rates (11, 12). These findings suggest that PCNL is a reliable option for managing renal stones in patients with impaired kidney function, particularly for those with large or complex stones. In contrast, the safety of the newer technique, URS, in CKD patients is less well-documented but shows promising potential. While URS is widely recognized for its minimally invasive nature and shorter recovery times, there is limited evidence specifically evaluating its long-term outcomes and safety profile in patients with compromised renal function (16, 17). In CKD patients, URS offers potential advantages due to its minimally invasive nature, shorter hospitalization, and lower risk of bleeding and renal parenchymal injury compared to PCNL (6, 28). Previous smaller studies have suggested that URS may preserve renal function in selected CKD patients, supporting its safety profile (16). However, the lower stone-free rate of URS relative to PCNL raises concerns about residual stones, which may predispose patients to recurrent obstruction and infection, ultimately compromising renal outcomes (17, 29, 30). Our study adds to the limited evidence by demonstrating that URS is comparable to PCNL in terms of long-term MAKE and survival in CKD patients. These findings suggest that URS may be considered a reasonable alternative, particularly in patients with high surgical risk, though further prospective studies are needed to confirm these benefits and address the impact of residual stone burden. This study leveraged a large multicenter database and PSM to enhance statistical power and minimize the impact of measured confounders. Our findings suggest that URS could be considered a less invasive alternative to PCNL for patients with CKD, particularly in cases where minimizing surgical risk is a priority. Future studies, particularly randomized controlled trials, are needed to validate these findings and explore long-term outcomes in CKD patients undergoing URS or PCNL.

### Limitation

This study has several limitations. First, its retrospective design may introduce inherent selection bias, residual confounding, and misclassification of exposures or outcomes, despite the application of PSM to balance measured covariates. Second, detailed clinical information such as stone size, composition, and location was not available in the database, which represents a major limitation given their strong influence on treatment outcomes. In addition, key procedural outcomes, including stone-free rate, retreatment rate, hospital stay, and procedure-specific complications, were not captured. Finally, technical variations, such as the use of suction or access sheaths during URS and ultrasound guidance or specialized devices during PCNL, were also unavailable, which may have further affected outcomes. Third, the database does not provide information on the size or location of the renal stones, which are important factors influencing treatment outcomes. Fourth, the event rate for dialysis was low, which may have reduced the statistical power to detect significant differences in this outcome. Third, as an observational study, it cannot establish causal relationships between the treatments and outcomes. Fifth, the TriNetX database does not provide sufficient detail to distinguish between rigid ureteroscopy and flexible retrograde intrarenal surgery. The inability to precisely differentiate procedure types remains a limitation.

Sixth, the database does not include information on the causes of death, limiting our ability to analyze mortality. Finally, being a retrospective study also makes the findings inherently weaker; therefore, we recommend that similar investigations be conducted prospectively to provide more definitive evidence.

## Conclusion

This study demonstrates that both URS and PCNL are viable options for managing renal stones in patients with CKD, with no significant differences observed in 5-year MAKE rates, all-cause mortality, or dialysis dependence between the two procedures. Given its minimally invasive nature and comparable long-term safety profile, URS can be considered a safe alternative to PCNL in CKD patients, particularly in those where reducing surgical risk is a priority. Further research is necessary to confirm these results and provide a deeper understanding of the long-term outcomes of URS and PCNL in CKD patients.

## Data Availability

Publicly available datasets were analyzed in this study. This data can be found at: https://trinetx.com/.
