# Supplementary material for: Comparative outcomes of ureteroscopy and percutaneous nephrolithotomy in CKD patients with renal calculi: a propensity-matched cohort study
Source: Front Med (Lausanne). 2025 Oct 3;12:1644526. doi: 10.3389/fmed.2025.1644526 (PMC12531238; doi:10.3389/fmed.2025.1644526)

Supplementary Table 1: Demographic, Diagnostic, and Laboratory Codes Used in the Definition of Covariates

| Category | Code | Description |
| --- | --- | --- |
| Demographics | AI | Age at Index |
| Demographics | M | Male |
| Demographics | F | Female |
| Demographics | 2106-3 | White |
| Demographics | 2186-5 | Not Hispanic or Latino |
| Demographics | 2054-5 | Black or African American |
| Demographics | 2028-9 | Asian |
| Demographics | UNK | Unknown race |
| Diagnoses | I10 | Essential hypertension |
| Diagnoses | E08-E13 | Diabetes mellitus |
| Diagnoses | E78 | Disorder of lipoprotein metabolism |
| Diagnoses | E66 | Overweight and obesity |
| Diagnoses | I20-I25 | Ischemic heart disease |
| Diagnoses | I50 | Heart failure |
| Diagnoses | I60-I69 | Cerebrovascular disease |
| Diagnoses | N21.0 | Calculus in bladder |
| Diagnoses | E21 | Hyperparathyroidism and disorder of parathyroid gland |
| Diagnoses | E21.3 | Hyperparathyroidism, unspecified |
| Diagnoses | M32 | Systemic lupus erythematosus |
| Diagnoses | 274.11 | Uric acid nephrolithiasis |
| Laboratory data | 8001 | Glomerular filtration rate |
| Laboratory data | 9022 | Calcium |
| Laboratory data | 9027 | Phosphate |
| Laboratory data | 9026 | Magnesium |
| Laboratory data | 2731-8 | Parathyrin, intact |
| Laboratory data | 9071 | Urate |
| Laboratory data | 32138-0 | Cystine |

Supplementary Table 2: Subgroup, and sensitivity analysis

|  | Hazard ratio | 95% CI |
| --- | --- | --- |
| Subgroup analysis | | |
| Age > 65 years old | 0.80 | 0.59-1.10 |
| Age < 65 years old | 0.77 | 0.37-1.60 |
| Male | 1.06 | 0.70-1.59 |
| Female | 0.90 | 0.56-1.42 |
| With diabetes mellitus | 0.90 | 0.57-1.41 |
| Without diabetes mellitus | 0.87 | 0.57-1.33 |
| With obesity | 0.89 | 0.54-1.47 |
| Without obesity | 0.94 | 0.63-1.40 |
| With heart failure | 0.96 | 0.59-1.56 |
| Without heart failure | 0.98 | 0.66-1.46 |
| With coronary artery disease | 0.86 | 0.48-1.53 |
| Without coronary artery disease | 0.92 | 0.65-1.31 |
| With advanced kidney disease | 1.75 | 0.76-4.00 |
| Sensitivity analysis | | |
| Follow up one year | 1.19 | 0.66-2.16 |
| Follow up three years | 1.08 | 0.71-1.63 |
| Result with no PSM | 1.01 | 0.81-1.25 |

Supplementary Figure legend

Supplementary Figure 1: the pre- and post-propensity score matching density curves


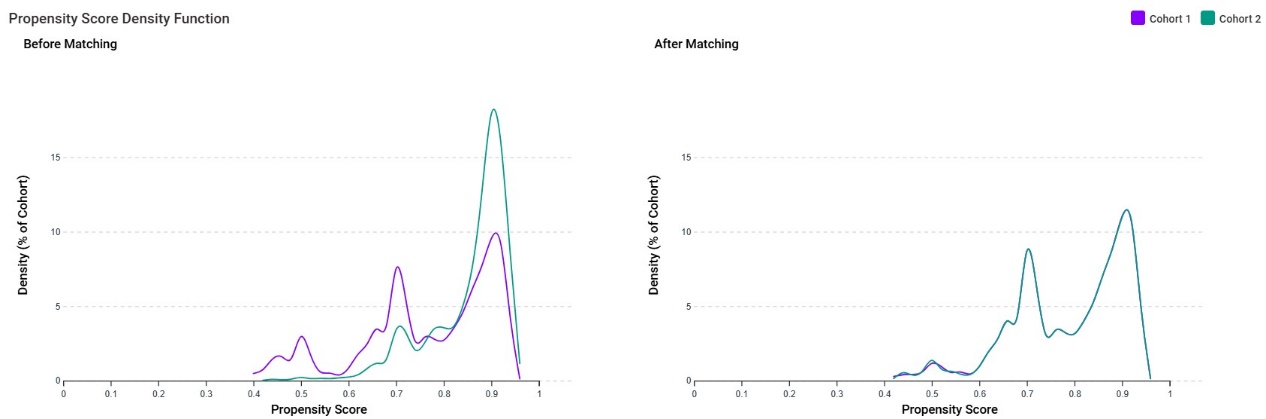

Supplement: Supplementary file 1 [file Data_Sheet_1.docx]
